# Fracture resistance after implantoplasty in three implant-abutment connection designs

**DOI:** 10.4317/medoral.23700

**Published:** 2020-07-19

**Authors:** Octavi Camps-Font, Albert González-Barnadas, Javier Mir-Mari, Rui Figueiredo, Cosme Gay-Escoda, Eduard Valmaseda-Castellón

**Affiliations:** 1 DDS, MS. Oral Surgery and Implantology, Faculty of Medicine and Health Sciences, University of Barcelona, Barcelona, Spain; 2 DDS, MS, PhD. Oral Surgery and Implantology, Faculty of Medicine and Health Sciences, University of Barcelona, Barcelona, Spain; 3 MD, DDS, MS, PhD, EBOS, OMF. Oral Surgery and Implantology, Faculty of Medicine and Health Sciences, University of Barcelona, Barcelona, Spain; 4 Oral Surgery and Implantology, Faculty of Medicine, Efhre International University, Belize City, Belize

## Abstract

**Background:**

To assess the effect of implantoplasty and implant-abutment design on the fracture resistance and macroscopic morphology of narrow-diameter (3.5 mm) dental implants.

**Material and Methods:**

Screw-shaped titanium dental implants (n = 48) were studied in vitro. Three groups (n = 16) were established, based on implant-abutment connection type: external hexagon, internal hexagon and conical. Eight implants from each group were subjected to an implantoplasty procedure; the remaining 8 implants served as controls. Implant wall thickness was recorded. All samples were subjected to a static strength test.

**Results:**

The mean wall thickness reductions varied between 106.46 and 153.75 µm. The mean fracture strengths for the control and test groups were, respectively, 1211.90±89.95 N and 873.11±92.37 N in the external hexagon implants; 918.41±97.19 N and 661.29±58.03 N in the internal hexagon implants; and 1058.67±114.05 N and 747.32±90.05 N in the conical connection implants. Implant wall thickness and fracture resistance (*P* < 0.001) showed a positive correlation. Fracture strength was influenced by both implantoplasty (*P* < 0.001) and connection type (*P* < 0.001).

**Conclusions:**

Implantoplasty in diameter-reduced implants decreases implant wall thickness and fracture resistance, and varies depending on the implant-abutment connection. Internal hexagon and conical connection implants seem to be more prone to fracture after implantoplasty.

** Key words:**Dental implants, narrow diameter, implant connection, peri-implantitis, implantoplasty, fracture strength.

## Introduction

Over recent decades, oral rehabilitation with dental implants has shown highly satisfactory results regarding restoration of the patient’s function and esthetics, with predicTable and safe long-term results (1,2). However, both short- and long-term complications may arise (3). In particular, peri-implantitis is becoming a more common condition, reaching a frequency close to 20% when determined at the patient level (4). Peri-implantitis is defined as a chronic inflammatory disease of an infectious nature characterized by peri-implant soft tissue inflammation in combination with radiographic bone loss (5). The main risk indicators associated with peri-implantitis are a history of chronic periodontitis, smoking, poor plaque control, and no regular maintenance care after implant therapy (6,7).

Different protocols have been suggested in the treatment of peri-implantitis. As non-surgical procedures alone appear to be insufficient to resolve these lesions, additional surgical treatment by means of open flap debridement combined with augmentative and/or resective approaches seems to be necessary (8).

Implantoplasty (IP) has been proposed as a procedure to smoothen and polish endosseous implant parts passing the bony envelope (9). The purpose of IP is firstly to remove the infected layer of titanium and secondly to produce a less plaque-retentive surface by reducing its roughness (10). Several case series and clinical trials have demonstrated a beneficial effect of IP when it is included as part of a resective surgical approach (11,12) or a combined resective and regenerative strategy (13-15). Nevertheless, IP reduces the implant diameter and the thickness of the implant wall and, together with the bone loss from peri-implantitis, may increase the risk of fixture fracture (16-18).

Both the diameter and type of implant-abutment connection design determine the thickness of the implant wall. Accordingly, the impact of IP on the mechanical properties could be greater in implants with a thinner wall. An *in vitro* study comparing standard (3.75 mm) and wide diameter implants (4.7 mm) concluded that IP reduced the strength of standard implants, but no changes occurred in wide fixtures (19). Similarly, Gehrke *et al*. (20) compared three different implant connections and found that the resistance to loading of 4 mm diameter implants decreased significantly after IP but varied among the three implant-abutment designs, the Morse tapered fixtures being the most resistant. However, none of these studies were conducted under the most unfavorable conditions (i.e. ≤3.5 mm diameter implants with a bone loss equivalent to 50% of their length). Therefore, the aim of the present study was to assess whether IP and implant-abutment design influence the fracture resistance of narrow-diameter, screw-shaped, rough-surfaced titanium dental implants in conjunction with a horizontal peri-implant defect corresponding to half of their length. The secondary objectives were to time the IP procedure and to assess the morphological changes that occurred in the treated areas.

## Material and Methods

An *in vitro* study was conducted using 48 tapered titanium grade 5 dental implants with similar macroscopic and microscopic designs (Biomimetic Ocean®, Avinent® Implant System, Santpedor, Spain). Three different implant-abutment connection designs were used for a total of 16 fixtures in each group: external hexagonal connection (EC), internal hexagonal connection (IC) and conical connection (CC). The platform and body diameters of the implants were 3.5 mm and the total body length was 10 mm. The implant threads were V-shaped and measured 0.08 and 0.28 mm in depth at the neck and body of the fixture, respectively.

The surface was moderately rough as a result of the sandblasting, acid-etching and anodizing techniques.

Using a computer generated random sequence, 8 implants per group received IP on the coronal half, and the remaining 8 served as controls in the fracture resistance tests.

- Cast preparation

All the implants were embedded and placed in exactly the same position in 3x1.7 cm epoxy resin casts (EA 3471 A and B Loctite®, Henkel AG & Company, Düsseldorf, Germany) with a Young’s modulus of elasticity (≥3GPa), in such a way that 5 mm of rough surface was exposed. This simulated a horizontal supracrestal peri-implant defect of 5 mm (50% of the total implant length), which is 2 mm more than the International Standardization Organization (ISO) guidelines.

- IP procedure

A cover screw was inserted to protect the implant’s connection from titanium debris. IP was performed manually by an experienced clinician (O.C-F.) using a handheld high-speed handpiece (GENTLEsilence LUX 8000B, KaVo® Dental GmbH, Biberach an der Riß, Germany) with abundant water cooling, under 2.8x magnification loupes with a LED light (Galilean HD and Focus™ LED 6000k, ExamVision ApS, Samsø, Denmark), and with adequate illumination in an environment similar to the dental setting. The pressure applied and number of strokes were not standardized to increase the external validity of the study. The operator’s other hand held and turned the cast.

The simplified 3-bur protocol described by Costa-Berenguer *et al*. (21) was followed. After removing the threads of the coronal half of the implants using an oval-shaped tungsten carbide bur (H379.314.023 Komet, GmbH & Co. KG, Lemgo, Germany), the surface was sequentially polished with two silicon carbide polishers (9618.314.030 and 9608.314.030 Komet, GmbH & Co. KG, Lemgo, Germany) until the operator felt that the exposed threads and structured areas were adequately smoothed. An external examiner (A.G-B.) registered the time employed with each bur. A new set of burs was used for each implant. Finally, the cover screw was removed.

- Macroscopic changes in implants

Periapical radiographs of all the implants were taken with a standardized long-cone paralleling technique. The digital images were used to assess the average thickness of the left and right implant walls at the implant shoulder (T0) and at 2.5 mm (T2.5) and 5 mm (T5) in an apical direction. The IP group measurements were subtracted from those of their control analogues to obtain the wall thinning calculation for each point. The six measurements were then repeated after turning the implant 120º and 240º, leading to a total of 18 measurements for each implant. This procedure was performed by the same trained examiner (A.G-B.) using ImageJTM software (National Institutes of Health, Bethesda, Maryland, USA). To test intraexaminer agreement and consistency, the assessment of 15 randomly selected radiographs (90 measurements) was repeated after 2 weeks. The intraclass correlation coefﬁcients (ICC) were 0.97 (95% confidence interval (95%CI) 0.94 to 0.99; *p*<0.001) and 0.98 (95%CI 0.96 to 0.99); *P*<0.001), showing excellent reliability and consistency.

- Static strength test

All the implants (n = 48) were subjected to a static compressive force. The tests were performed according to the specifications of the ISO 14801:2016 guideline, except for the defect size.

Identical metal hemispherical load abutments (n = 48) were digitally designed, milled and placed on each implant. After applying a 32 N·Cm torque, prosthetic screws (Avinent® Implant System, Santpedor, Spain) were used to retain the load abutments.

All the tests were performed at a constant speed of 1 mm/min with a MTS Bionix 370 Load Frame universal servo-hydraulic mechanical testing machine (MTS®, Eden Prairie, USA), applying a compression force to the implants with a MTS Load Cell 661.19H-03 of 15kN capacity. All the samples were held with the same device, a manufactured stainless-steel clamping jaw that allowed compression forces to be applied at a constant angle of 30º from the vertical axis. The tests were controlled with TestStar II® software (MTS®, Eden Prairie, USA), which recorded real-time data from the 48 samples. The maximum compression force (Fmax) was measured as the maximum force reached until the sample fractured or exhibited a significant amount of plastic deformation and a load drop before implant fracture.

- Surface evaluation and fractographic analysis

All samples were assessed by scanning electron microscopy (SEM; Quanta-200, Field Electron and Ion Company, Hillsboro, USA) to determine their surface quality semi-quantitatively, as well as the presence of impurities, fractures, deformation, scuffing, cracks, or fissures. Images of the fractured components were also taken.

- Statistical analysis

The sample size calculation was based on results reported by Gehrke *et al*. (20) Considering Fmax as the primary outcome measure, the expected mean reductions in fracture resistance after IP were 286 N, 333.7 N and 180.5 N in the EC, IC and CC groups, respectively, with a common standard deviation of 100 N. Under these assumptions, 8 implants per group were required (two-way analysis of variance (ANOVA), α = 0.05 and 1-β = 0.80).

The implant characteristics were presented as absolute and relative frequencies for categorical outcomes. Normality of scale variables (Fmax and implant wall thickness) were explored through Shapiro-Wilk’s test and visual analysis of the P-P and box plots. Where normality was rejected, the interquartile range (IQR) and median were calculated. Where the distribution was compatible with normality, the mean and standard deviation (SD) were used.

To analyze the effect of the procedure (IP or control), implant-abutment design (EC, IC or CC) and the interaction between these two variables on Fmax, a two-way ANOVA was performed. Fulfillment of the assumptions was ensured through tests of normality and homogeneity of variances (i.e. Shapiro-Wilk’s and Levene’s tests, respectively). For each procedure and implant-abutment design, pairwise comparisons between groups were performed.

An unpaired t test was used to identify differences in implant wall thickness between the control and IP groups at every reference point. Differences in thickness between the implant-abutment designs at each of the 3 reference points were assessed by means of a one-way ANOVA. For each area of interest, Pearson correlation coefficients were computed to quantify the correlation between the implant wall thickness and Fmax.

The association of categorical variables was assessed with either Pearson’s 2 test or Fisher’s exact test.

The statistical analysis was carried out with Stata14 (StataCorp®, College Station, TX, USA). The level of significance was set at *P* < 0.05, using Tukey’s correction for multiplicity of contrasts.

## Results

All samples were treated without registering any deviation from the protocol.

- Treatment time

The mean time used with the carbide burs was 5 min 32 s (SD = 35 s), and the two silicon carbide polishers were employed for mean times of 2 min 41 s (SD = 20 s) and 2 min 25 s (SD = 26 s), respectively. The total IP mean time per implant was 10 min 37 s (SD = 55 s).

- Macroscopic changes to implants

The mean reductions in implant wall thickness after IP are summarized in Table 1. In all groups, the procedure was associated with a statistically significant reduction in thickness (*P* ≤ 0.05; unpaired t test) at T0-5. The amount of change was similar in all the implant-abutment designs at each reference point (all *P* > 0.05; one-way ANOVA).

**Figure 1:**
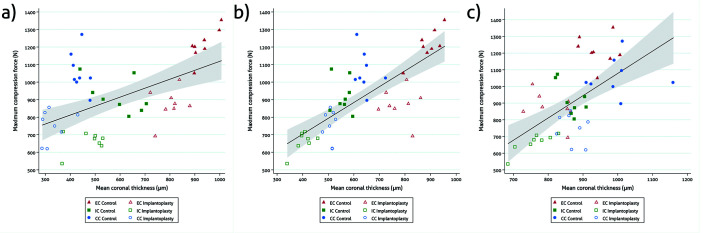
Scattergrams with a 95%CI showing the correlation between coronal (a), middle (b) and apical (c) implant wall thickness and fracture resistance.

There was a positive correlation between implant wall thickness and Fmax at T0 (r = 0.55; n = 48; *P* < 0.001), T2.5 (r = 0.75; n = 48; *P* < 0.001) and T5 (r = 0.61; n = 48; *P* < 0.001) (Fig. 1).

Perforations of the implant wall were not observed.

- Static strength test

Compression tests revealed that IP produced a significant reduction in fracture resistance (F[1, 42]=130.31, *P* < 0.001). Specifically, the EC, IC and CC groups experienced a Fmax decrease of 27.96%, 28.00% and 29.41%, respectively (Table 2).

**Table 1:**
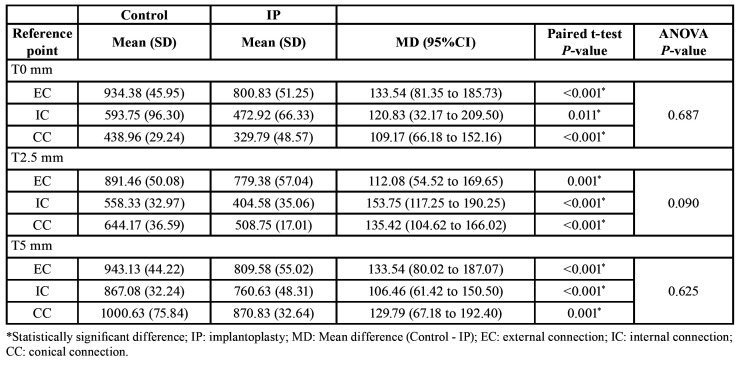
Mean implant wall thickness (µm) in IP and control samples at each reference point (n=48).

**Table 2:**
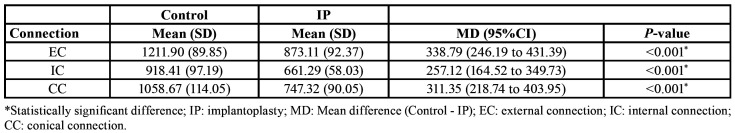
Mean fracture strength (N) of the three implant-abutment designs in IP and control samples.

Fmax was also influenced by the implant connection design (F[2, 42)]= 30.43, *P* < 0.001). In both the control and IP samples, the EC implants were more resistant than the IC [(MDcontrol = 293.49 N; 95%CIcontrol = 182.00 to 404.97; t[42]control = 6.40; Pcontrol < 0.001) (MDIP = 211.82 N; 95%CIIP = 100.34 to 323.30; t[42]IP = 4.62; PIP < 0.001)] and CC [(MDcontrol = 153.23 N; 95%CIcontrol = 41.75 to 264.71; t[42]control = 3.34; Pcontrol = 0.005) (MDIP = 125.79 N; 95%CIIP = 33.18 to 218.39; t[42]IP = 2.74; PIP = 0.024)] fixtures. Among the control samples, the CC implants withstood significantly higher compression forces than the IC ones (MD = 140.26 N; 95%CI = 28.74 to 251.74; t[42] = 3.06; *P* = 0.012) (Table 3). However, the results for both CC and IC implants were similar after IP (Table 3).

The effect of interaction between implant-abutment design and procedure was not significant (F[2, 42] = 0.82, *P* = 0.447).

- Surface evaluation and fractographic analysis

**Figure 2:**
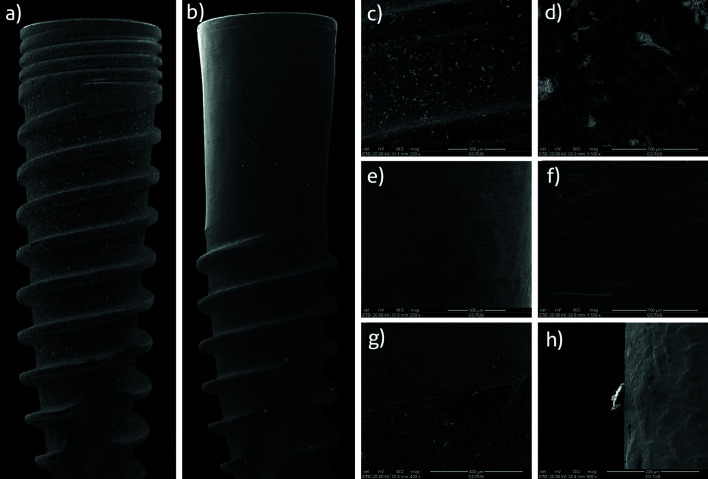
Scanning electron microscopy (SEM) macrographs of a control (a) and a test (b) implant. Detail of a control implant surface at 200 × (c) and 1500 × (d) magnification with SEM. Detail of a test implant polished surface without any debris at 200 × (e) and 1500 × (f) magnification with SEM. Detail at 200 × magnification of the interface between the polished and untreated portion of a test implant (g). Detail at 800 × magnification of silicon debris found on the polished area of a test implant (h).

SEM analysis of the control implants showed a good quality surface finish, free of fissures, and/or defects from the machining process. The surface was moderately rough as a result of the sandblasting, acid-etching and anodizing techniques (Fig. 2).

IP samples showed a smooth surface, free of grooves. Titanium shavings were identified in the areas of interest. Silicon debris was also found in some of the samples (Fig. 2).

**Figure 3:**
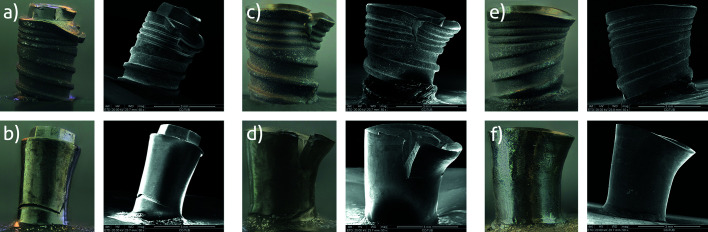
(a) Control EC implant fractured at the implant-abutment connection; (b) Test EC implant fractured through the body; (c, d) Control and test IC implants fractured in the cervical area; (e, f) Control and test CC implants fractured at the implant-abutment connection.

Two fracture patterns were identified: 1) perpendicular to the longitudinal axis of the implant, with fracture of the implant platform or body (Fig. 3), and 2) through the implant-abutment connection with fracture of the abutment screw (Fig. 3).

Overall, 20 control specimens (83.3%) fractured through the abutment screw, while 16 IP samples (66.7%) fractured through the implant (2[1] = 12.34; *P* < 0.001). Deformation at the edge of the implant platform on the opposite side to the application of force was observed in 89.6% (n = 43) of cases.

SEM analysis of the implant fractures showed that the breakage was located where the threads and the implant body had the lowest cross-sectional thickness. These were ductile fractures with plastic deformation.

The fracture pattern recorded for each implant connection design is described in Table 4.

**Table 3:**
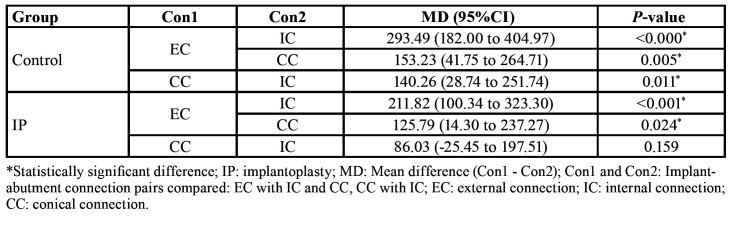
Differences in mean fracture strength (N) of the IP and control samples for the three implant-abutment connections.

**Table 4:**
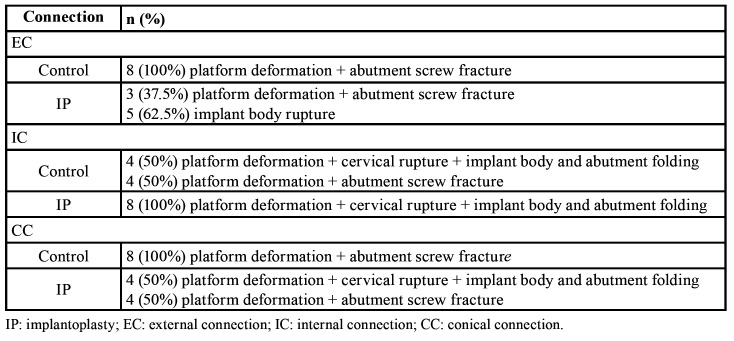
Fractographic analysis by implant abutment-design (n=48).

## Discussion

The present *in vitro* study aimed to describe and analyze the influence of a specific IP protocol on the fracture resistance and wall thickness of narrow-diameter implants in three implant-abutment designs.

Marginal bone loss associated with peri-implantitis usually extends to the level corresponding to the end of the internal chamber of the abutment screw, where resistance to bending is diminished (18,22,23). Gehrke *et al*. (17) reported average reductions in strength of 37.2% and 53.8% when the level of cervical insertion was located 3 mm and 5 mm below the implant shoulder, respectively. Consequently, the samples in the present study were manufactured to simulate a horizontal peri-implant defect of 5 mm (50% of the total implant length) in order to recreate a common but mechanically unfavorable situation (18).

Although a recent investigation using EC standard-diameter implants concluded that IP does not seem to decrease the fracture strength (21), others have revealed a weakening of the fixture when IP is performed (18-20). The latter is in accordance with the present observations, where IP produced an almost 30% decrease in fracture resistance (F[1, 42] = 130.31, *P* < 0.001) (Table 2). This weakening also implied a change in the fracture pattern, since, while 20 of the 24 control specimens (83.33%) fractured through the abutment screw, the rupture site of the majority of the test samples (n = 16; 66.67%) was in the platform or body (2[1] = 12.34; *P* < 0.001). This suggests that a certain implant wall thickness is required in order to resist bending forces (19).

Fracture resistance was also influenced by implant abutment-design (F[2, 42] = 30.43, *P* < 0.001) (Table 3). Basically, it was observed that in both the control and the IP samples, the EC implants were the least prone to fractures whereas the IC implants were the most susceptible to rupture. Conversely, Gehrke *et al*. (20) found that Morse tapered fixtures presented significantly greater resistance than EC and IC ones. Differences in the IP protocol applied (mechanical lathe machine vs. manual simplified 3-bur protocol), and the implant dimensions used (4x11 mm vs. 3.5x10 mm), as well as the macroscopic design of the fixtures, may explain these conflicting results. Hence, future studies should be performed with implant designs and IP protocols differing from the aforementioned in order to generalize the present results. In addition, considering that masticatory forces are cyclic, dynamic fatigue tests should be carried out to predict how long the implants will function properly.

In accordance with ISO 14801:2016, the smallest diameter implant was used in order to simulate the most unfavorable clinical scenario. In this context, to the best of the authors’ knowledge, the present report is the first that assesses the effect of IP on narrow-diameter implants. Previous *in vitro* studies and finite element analyses have illustrated that stress values affecting the crestal cortical bone are reciprocal to the dental implant diameter, thus resulting in disadvantageous stress peaks at the implant-bone interface and a higher risk of fatigue fracture (24). The average maximum bite force for adults in the posterior regions is 847 N for men and 597 N for women (25). In the present study, the fracture strengths after static loading of the control specimens were above these thresholds (Table 2). However, when IP was performed, all three groups showed Fmax values close to those masticatory forces (Table 2). Accordingly, the clinician should be cautious when applying this procedure to narrow-diameter implants, especially those with an internal connection, at least until human studies shed some light on this topic.

Generally, implant strength is derived from the thickness of the implant wall (19,26,27), which, in turn, is determined by the implant-abutment connection design. For this reason, we decided not to use the thickness of the implant wall as a possible confounding factor due to the existence of collinearity between the two variables and the difficulty of measuring the actual reduction of the implant wall clinically. The present results agree with previous publications, since a statistically significant positive correlation with implant wall thickness was found at each of the reference points assessed (Fig. 1). It should be noted that the walls of the IC and CC implants at T0 and T2.5 were almost half the width of the walls of the EC implants (Table 1). As a result, the EC implants had significantly higher Fmax values than the IC and CC implants in both the control and the IP samples. Indeed, the mean Fmax values obtained in the EC group after IP were similar to those observed in the control IC implants. In addition, the thickness of the wall was also related to the fracture pattern, since implant rupture occurred in the portion where the wall was thinnest.

As any *in vitro* study, a possible limitation of the present report is that IP was performed under ideal conditions. In an environment closer to a real clinical situation, the results could differ from those obtained in this study. Another potential drawback is related to the fact that IP was performed manually. This leads to a lack of standardization of important variables like pressure and number of times that the burs were applied to the implant surface (21). On the other hand, this procedure is more similar to a real clinical scenario, and therefore increases the external validity of the study.

The simplified 3-bur IP protocol employing an average of 11 min (SD = 1) was able to produce a minimal implant diameter reduction, restricted to the threads and without affecting the internal diameter of the specimens. These outcomes were not influenced by the implant connection design (Table 1). In this context, it must be emphasized that a potential drawback of the present study was the lack of further analyses to evaluate the surface roughness of the smoothed implants. However, a recent report employing the same IP protocol has shown a mean Sa value of 0.1 µm (SD = 0.02), which is significantly lower than the 0.76 µm (SD = 0.08) of the untreated controls (24). Similarly, Ramel *et al*. (10) obtained an effective smoothening of the surface through an IP procedure combining 3 diamond burs and 2 silicone polishers which took 21 minutes (SD = 4) per implant. Nevertheless, the method they applied resulted in an average Ra value of 0.32 µm (SD = 0.14), which is higher than the Ra threshold (< 0.2 µm) beyond which bacterial adhesion cannot be further reduced (28-30). Future research should determine the most efficient, effective and safest IP procedure.

## Conclusions

Implantoplasty in diameter-reduced implants produces a decrease in fracture resistance in all the narrow-diameter titanium dental implant groups tested. The implant-abutment connection design influences the resistance of the fixture. Clinicians should be aware that implantoplasty might increase the risk of fracture, especially in narrow-diameter internal connection implants (CC and IC).
